# The Role of Outer Membrane Protein 16 in *Brucella* Pathogenesis, Vaccine Development, and Diagnostic Applications

**DOI:** 10.3390/vetsci12070605

**Published:** 2025-06-20

**Authors:** Lu Zhang, Jun Bai, Long Li, Yanqing Jia, Xinxin Qiu, Yan Luo, Dong Zhou, Zhencang Zhang

**Affiliations:** 1 Department of Animal Engineering, Yangling Vocational & Technical College, Yangling 712100, China; luzhang2021@nwafu.edu.cn (L.Z.); wshbj@163.com (J.B.); lilong1101@126.com (L.L.); yqjia1987@163.com (Y.J.); ylzyqiuxinxin@163.com (X.Q.); yl_ly2019@163.com (Y.L.); 2 Shaanxi Engineering Research Center of the Prevention and Control for Animal Disease, Yangling Vocational & Technical College, Yangling 712100, China; 3 Key Laboratory for Efficient Ruminant Breeding Technology of Higher Education Institutions in Shaanxi Province, Yangling Vocational and Technical College, Yangling 712100, China; 4 Yangling Vocational and Technical College, The Youth Innovation Team of Shaanxi Universities, Yangling 712100, China; 5 Shenzhen Research Institute, Northwest A&F University, Shenzhen 518000, China; 6 College of Veterinary Medicine, Northwest A&F University, Yangling 712100, China

**Keywords:** *Brucella*, outer membrane, vaccine, diagnose

## Abstract

Brucellosis, caused by *Brucella* species, remains a significant zoonotic disease affecting both human and animal health worldwide. Among the outer membrane proteins (Omps) of *Brucella*, Omp16 has emerged as a key immunogenic target with potential applications in vaccine development and diagnostics. Omp16, a lipidated peptidoglycan-associated lipoprotein, stimulates a strong proinflammatory response and is essential for maintaining the bacterial outer membrane integrity and facilitating host cell invasion. This review examines the immunogenic properties of Omp16, its role in *Brucella* pathogenesis, and its potential as a candidate for vaccine development. We discuss how Omp16-based vaccines, including recombinant proteins, outer membrane vesicles, and viral vector vaccines, have shown promise in providing protection against *Brucella* infections in animal models. Additionally, Omp16’s utility in diagnostic applications, particularly in enzyme-linked immunosorbent assays (ELISA), offers a reliable method for detecting brucellosis in both humans and animals. Overall, Omp16 represents a crucial antigen with significant potential for advancing both the diagnosis and prevention of brucellosis, offering insights into the next generation of brucellosis vaccines and diagnostic tools.

## Simple Summary

Brucellosis is a widespread disease that affects both animals and humans, leading to serious health and economic problems. Finding better ways to detect and prevent this disease is critical. This review focuses on a protein called outer membrane protein 16 (Omp16), which is found in *Brucella* bacteria. Omp16 plays important roles in helping the bacteria survive in the host, trigger immune responses, and possibly protect against infection. Scientists have also explored Omp16 as a tool for developing new vaccines and diagnostic tests. Although it shows promise, more research is still needed to confirm its reliability and effectiveness, especially when compared with other well-studied proteins. This article provides a summary of current findings about Omp16 and discusses its potential and limitations in *Brucella* research.

## 1. Introduction

*Brucella* species are facultative intracellular pathogens responsible for brucellosis, one of the most prevalent zoonotic diseases worldwide. The disease is primarily characterized by reproductive failures such as abortion, infertility, and placentitis in animals, which have significant economic implications due to the loss of livestock productivity [1,2]. Transmission to humans occurs primarily through direct contact with infected animals or consumption of contaminated animal products [3,4]. Brucellosis manifests as a febrile illness with symptoms including fever, sweats, malaise, and musculoskeletal pain in humans. It is estimated that more than 500,000 new human cases of brucellosis occur annually [5].

Brucellosis encompasses a group of infectious diseases primarily caused by facultative intracellular bacteria belonging to the genus *Brucella*. While multiple *Brucella* species exist, this review focuses specifically on brucellosis caused by the classic, highly zoonotic species responsible for the vast majority of human infections globally: *Brucella melitensis*, *Brucella abortus*, *Brucella suis*, and *Brucella canis*. Other species, such as *Brucella ovis* and *Brucella neotomae*, primarily infect animals and pose negligible zoonotic risk, while the zoonotic potential of marine mammal-associated strains like *Brucella ceti*, *Brucella pinnipedialis* remains less common. Most recently, a group of taxonomists merged the *Brucella* with the primarily free-living, phylogenetically related *Ochrobactrum* spp. in the genus *Brucella*, but it is still undetermined [6,7,8,9]. The pathogen’s ability to evade the host immune system and persist within macrophages complicates treatment and eradication efforts, making brucellosis a challenging public health issue, particularly in regions where animal husbandry is prevalent [10,11].

**Figure 1:**
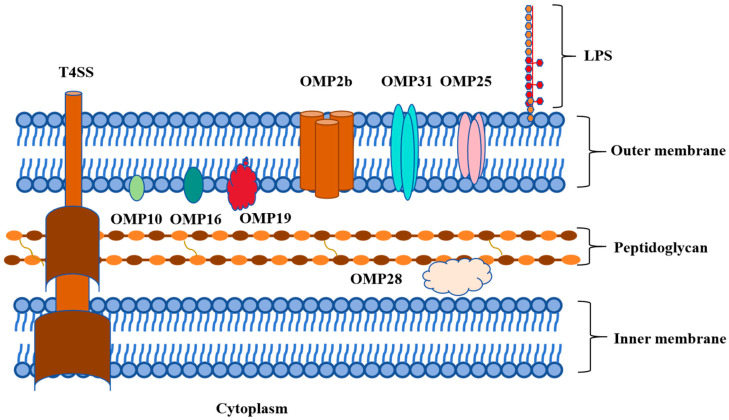
Graphical abstract of outer membrane proteins of *Brucella*.

Outer membrane protein is the main component of the cell wall of *Brucella*, which has strong immunogenicity and protection, and is related to the virulence of the bacteria [12]. The outer membrane proteins of *Brucella* can be divided into three groups according to their molecular weights. The first group of outer membrane proteins, consisting of 10 KDa (Omp10), 18 KDa (Omp16), and 19 KDa (Omp19), are lipoproteins that are closely related to *Brucella* virulence. The second group of outer membrane proteins, including Omp22, Omp25, Omp25A, Omp25B, Omp25C, Omp25D, and Omp31, weighing 25–27 KDa and 31–34 KDa, respectively, play important roles in maintaining the integrity of the cell envelope (Figure 1). These Omps are pore proteins that conjunct with the LPS O-chain to protect *Brucella* from complement and other antimicrobial peptides encountered in the host. The third group of Omps, comprising Omp2a and Omp2b, possess a molecular weight ranging from 36 to 38 KDa and are homologous outer membrane proteins that exhibit properties characteristic of bacterial porins [13,14]. Omps are important virulence factors of *Brucella*. Furthermore, they act as pathogen-associated molecular patterns (PAMPs) to activate receptors activating the immune response.

Omp16 is essential for *Brucella* survival, as its deletion impairs the integrity of the outer membrane and reduces bacterial viability in vitro [15,16]. Omp16 is a key mediator of the inflammatory response and a self-adjuvanting antigen based on its ability to stimulate dendritic cells and promote a Th1-biased immune response without the need for external adjuvants [17].

Given the significant impact of *Brucella* infections on human health and livestock productivity, understanding the molecular mechanisms underlying *Brucella* pathogenesis is crucial for developing effective control measures. This review aims to provide a comprehensive overview of the current knowledge on Omp16, focusing on its role in *Brucella* pathogenesis. Additionally, the review will address the challenges associated with using Omp16 as a diagnostic marker or vaccine antigen and suggest potential strategies to overcome these hurdles.

## 2. Structural and Functional Characteristics of Omp16

The *Omp16* gene, located on chromosome I of *Brucella*, is 507 bp in length and encodes a 168-amino acid protein with a molecular weight of approximately 16 kDa. Omp16 is homologous to the peptidoglycan-associated lipoprotein (Pal) and contains the characteristic Pal domain. It is highly conserved across multiple *Brucella* species, including *Brucella abortus*, *Brucella melitensis*, *Brucella suis*, *Brucella canis,* and *Brucella ovis* [15]. An Omp16 interacts with the Tol–Pal system and plays a crucial role in maintaining the structural stability and functionality of the outer membrane. Although current evidence supports its presence in several representative *Brucella* species, further investigation is needed to confirm its universality across all known *Brucella* species [15,18].

On the genetic level, research into the essentiality of Omp16 reveals that attempts to generate Omp16-deficient mutants in *B. ovis* have been unsuccessful, indicating that Omp16 is essential for bacterial survival [16]. This contrasts with other Omps, such as Omp10 and Omp19, whose single deletions are viable. Such findings highlight Omp16’s critical role in the structural and functional integrity of *Brucella*’s outer membrane, contributing to its resilience and pathogenicity [16]. Further functional analysis of Omp16 reveals its role in inflammatory responses. Specifically, the lipidated form of Omp16 has been shown to stimulate the production of pro-inflammatory cytokines such as TNF-α, IL-6, IL-10, and IL-12 in host macrophages, distinguishing its inflammatory mechanism from that of lipopolysaccharides (LPS) [19]. A study indicates that unlipidated Omp16 (U-Omp16) could constitute a new PAMP recognized by TLR4 [17].

Furthermore, Omp16, found within the outer membrane vesicles of *Brucella* strains such as *B. suis*, *B. melitensis*, *B. ovis*, and *B. canis*, can modulate immune responses. The Outer Membrane Vesicles (OMVs) from both smooth and rough strains of *B. melitensis* were shown to induce pro-inflammatory cytokines like TNF-α and IL-6, while also inhibiting PD-L1 expression on T-cells, suggesting a complex immunomodulatory role [20]. Moreover, OMVs from rough mutant *B. melitensis* VTRM1 (lacking the side O chain of LPS) induced significantly higher expression of IL-12, TNF-α, and IFN-γ genes in bone marrow dendritic cells than OMVs from smooth *B. melitensis* 16 M [21]. Additionally, OMVs from rough-mutant strains demonstrated higher sensitivity to enzymatic and detergent treatments, implying a protective role of complete lipopolysaccharide (LPS) in OMVs from smooth strains [22]. The unique composition of OMVs, including the presence of Omp16, contributes to *Brucella*’s ability to evade the immune system while maintaining a robust response to pathogens.

The immunogenic properties of Omp16 have been the focus of numerous studies aimed at understanding the host immune response to *Brucella* infection and developing potential diagnostic and vaccine candidates. Omp16 is recognized by the host immune system as a PAMP, leading to the activation of both innate and adaptive immune responses [19,23].

Interestingly, the U-Omp16 exhibits distinct immune-modulating properties, inducing a Th1-skewed immune response that is critical for protective immunity against intracellular pathogens like *Brucella* [17]. In vitro studies revealed that immunization with U-Omp16 formulated with IFA induced a Th1-biased immune response and conferred protection against *B. abortus* infection comparable to that of the live S19 vaccine in mice. In vivo depletion of either CD4^+^ or CD8^+^ T cells abolished the protective effect, suggesting that both subsets contribute to Omp16-mediated immunity. While some experimental groups showed variable statistical significance, the overall trend supports the potential of U-Omp16 as a protective subunit antigen [23]. These antigen-specific T cells confer significant protection, as demonstrated by in vivo depletion experiments showing that both cell types are essential for immune protection.

The recombinant U-Omp16 protein was also effective in promoting a systemic and mucosal immune response. This was evidenced by increased IgG2a levels and IFN-γ production in splenocytes of immunized mice [24,25]. Such responses were comparable to those elicited by live attenuated *Brucella* vaccines like S19 and RB51, highlighting U-Omp16’s potential as a safer alternative to live vaccines [23].

Several studies have explored the use of Omp16 in vaccine formulations, including its incorporation into OMVs and recombinant platforms. OMVs from *Brucella melitensis* strains, enriched with Omp16 and other immunogenic proteins, induced robust IL-12, TNF-α, and IFN-γ responses in bone marrow-derived dendritic cells [21]. In animal models, OMVs provided significant protection against virulent *B. melitensis* challenges, with protection levels comparable to live attenuated vaccines. Notably, OMVs from rough strains elicited higher cell-mediated immunity, as evidenced by increased serum IgG2a levels and enhanced T-cell responses, compared to OMVs from smooth strains [23,26].

Recombinant platforms expressing Omp16, such as *Lactobacillus casei* constructs, have shown promising results in inducing humoral, cellular, and mucosal immunity. Mice immunized with recombinant *L. casei* expressing Omp16 demonstrated elevated IgG, IgA, and IFN-γ levels, indicating a comprehensive immune response [27]. These findings suggest that Omp16 not only acts as an effective antigen but also possesses self-adjuvanting properties that enhance vaccine efficacy.

Beyond its role in brucellosis, Omp16 has demonstrated potential in modulating immune responses in other contexts. For instance, sublingual administration of U-Omp16 with cow’s milk proteins suppressed allergic responses in murine models by promoting a Th1 immune profile and reducing IgE-mediated hypersensitivity [28]. Similarly, Omp16 dampened allergic symptoms and inhibited IL-5 production in the gastrointestinal tract, further supporting its utility as an immune-modulating agent [24,29].

## 3. Omp16 in Vaccine Development

Brucellosis control relies not only on coordinated eradication campaigns that include test-and-slaughter policies, movement restrictions, and surveillance strategies in endemic regions but also on vaccination. Brucellosis vaccines have primarily been developed and tested in murine models before being applied to natural host species such as cattle, sheep, goats, and pigs. The *Brucella abortus* S19 vaccine, originally developed in cattle and widely validated in mice, is used in calves to prevent bovine brucellosis caused by *B. abortus* [30]. It offers strong protection but may cause abortion in pregnant animals and interferes with serological testing. The *B. melitensis* Rev.1 vaccine, developed in goats and sheep, is the standard for controlling *B. melitensis* infection in small ruminants [31]. It provides effective protection but also carries a risk of abortion and persistence in tissues. The *Brucella suis* S2 vaccine, developed from a strain of *B. suis*, has been adapted for use in sheep, goats, and pigs in China, and offers oral administration with moderate protection and reduced virulence [32]. Rough vaccines such as *Brucella abortus* RB51 were derived through laboratory attenuation and initially evaluated in mice, then extensively used in cattle to target *Brucella abortus*, with the advantage of not interfering with serological diagnosis [33]. Inactivated vaccines like *Brucella abortus* 45/20 were also tested in rabbits and cattle and provided early alternatives for bovine brucellosis prevention, though their inconsistent efficacy has limited widespread adoption [34]. In recent years, recombinant and marker vaccines such as M5-90Δbp26 and A19ΔvirB12, first tested in mice and later applied in cattle or small ruminants, have shown potential for improved safety and diagnostic compatibility while targeting *Brucella abortus* or *Brucella melitensis* depending on the host species [35,36]. Overall, most current vaccines have originated from studies in mice, and their application varies by animal host and target *Brucella* species.

*Brucella* harbors a wide array of outer membrane proteins (Omps), including Omp19, Omp25, and Omp31, many of which contribute to its pathogenesis and have been investigated as vaccine antigens. However, Omp16 stands out as a particularly promising target due to its high conservation across all six classical *Brucella* species, strong immunogenic potential, and favorable safety profile in experimental studies. Unlike live attenuated vaccines that carry the risk of inducing abortion in pregnant animals, Omp16-based vaccines—especially in the form of recombinant proteins or multi-epitope constructs—do not contain viable organisms and have demonstrated no reproductive toxicity in preclinical mouse models. For example, MEV-Fc, a fusion vaccine incorporating Omp16, Omp19, Omp25, and L7/L12 epitopes, has shown strong protective immunity without any observed abortion or tissue pathology. These features make Omp16 an ideal candidate for inclusion in next-generation subunit vaccines, especially for use in pregnant or breeding livestock and potentially human applications.

Outer membrane protein 16 has emerged as a promising candidate for the development of vaccines against *Brucella* due to its ability to induce both humoral and cellular immune responses. Numerous studies have demonstrated that Omp16 is highly immunogenic and plays a crucial role in the pathogenesis of *Brucella* infections (Table 1). The protein has been shown to be present in *Brucella* OMVs and as a standalone recombinant antigen, both of which exhibit significant protective properties when used in vaccination strategies. OMVs from *Brucella melitensis,* enriched with Omp16, have been shown to induce strong immune responses, including elevated levels of TNF-α, IL-12, and IFN-γ, suggesting that Omp16 contributes to a Th1-biased immune response [21,22].

The lipidation status of Omp16 further influences its immunogenicity. L-Omp16 induces a robust pro-inflammatory cytokine profile in vitro and enhances cell-mediated immunity in vivo. Vaccination with L-Omp16 formulated with adjuvants like incomplete Freund’s adjuvant has been shown to provide protective immunity against *B. abortus* infections, eliciting high levels of IgG2a and IFN-γ, indicative of a strong Th1 response [23]. Interestingly, unlipidated Omp16 (U-Omp16) has demonstrated similar protective efficacy, with studies showing that oral or systemic administration of U-Omp16 induces comparable levels of protection to those seen with live vaccines [23]. U-Omp16 also exhibits self-adjuvanting properties, activating dendritic cells and macrophages to promote a protective immune response via TLR4 signaling, further supporting its potential as a vaccine candidate [17,19]. Immunization with a combination of four recombinant *Brucella abortus* proteins—Omp16, Omp19, Omp28, and L7/L12—induced a strong T helper 1 (Th1) immune response in BALB/c mice, characterized by high levels of IFN-γ, TNF-α, and IgG2a, and a predominance of pro-inflammatory cytokines [45].

The influenza viral vector vaccine expressing Omp16 has shown good immunogenicity in preventing brucellosis. Studies demonstrate that an influenza viral vector vaccine, such as Flu-BA, expressing Omp16, induces a strong T-cell immune response in pregnant cattle, sheep, and goats, significantly reducing *Brucella* infection rates and tissue colonization. Animals vaccinated with Flu-BA exhibited high levels of IgG2a antibodies and IFN-γ responses specific to *Brucella* Omp16, indicating effective activation of a Th1-type immune response. Notably, in pregnant ewes and does, the Flu-BA vaccine prevented abortion and provided good protection against *B. melitensis* infection, comparable to or exceeding the protection provided by the commercial *B. melitensis* Rev.1 vaccine [37,38,39,40,41,42,43,46,47,48]. Moreover, studies also suggest that the expression of Omp16 through the influenza vector maintains long-lasting immune protection, showing good safety and immunogenicity, particularly in small ruminants, confirming its potential as a promising candidate for brucellosis vaccination [37,49].

Additionally, recombinant vaccines expressing Omp16 have shown promise. Studies involving *Lactobacillus casei* expressing Omp16 have demonstrated the ability of this recombinant platform to stimulate both systemic and mucosal immunity, resulting in high levels of IgG, IgA, and IFN-γ production. The *L. casei*-Omp16-PEDVS recombinant vaccine, which expresses both PEDVS from PEDV and Omp16 from *Brucella abortus*, effectively induced elevated levels of IgG, neutralizing antibodies, IL-4, IL-10, and IFN-γ in serum, along with IgA in the feces of immunized mice [27]. *Brucella melitensis* Omp16 protein fused to the human interleukin 2 in *Lactococcus lactis* MG1363 induced a strong IgG immune response in BALB/c mice [50,51]. This highlights the versatility of Omp16 in various vaccine formulations, further emphasizing its potential in the development of a safe and effective vaccine against brucellosis [52]. The divalent DNA vaccine encoding both *Brucella abortus* L7/L12 and Omp16 proteins (pcDNA3.1-L7/L12-Omp16) induced robust humoral and cellular immune responses in BALB/c mice, with a dominant IgG2a response and a strong Th1-driven immune profile [44].

Omp16’s immunogenic properties, including its ability to stimulate a Th1 immune response, along with its effectiveness in various vaccine platforms, such as influenza viral vectors, OMVs, and recombinant antigens, position it as a key candidate for future *Brucella* vaccine development.

## 4. Diagnostic Applications of Omp16 in Brucellosis Detection

Outer membrane protein 16 has been widely studied for its potential use in diagnostic assays for brucellosis. Given its significant role as an immunogenic protein in *Brucella*, Omp16 has been explored as a diagnostic antigen in various serological tests, particularly enzyme-linked immunosorbent assays (ELISA). The development of a diagnostic indirect ELISA using recombinant Omp16 as the antigen has shown great promise. This test, designed for human brucellosis detection, was reported to exhibit 100% sensitivity and 95% specificity; however, it is important to note that the study included only a limited number of RBPT-positive samples, which may affect the generalizability of the results [53]. Although Omp16 has demonstrated immunogenicity, its diagnostic value appears limited when compared to other outer membrane proteins. For instance, Bai et al. found that BP26 and Omp31 outperformed Omp16 in terms of diagnostic accuracy across human and animal samples [54]. Similarly, Yao et al. reported that combinations of Omps, particularly Omp25/Omp31/BP26 and Omp31/BP26, provided improved sensitivity and specificity in detecting brucellosis across species [55]. Additionally, rOmp16-based ELISA demonstrated strong agreement with commercial IgG ELISA kits, making it a reliable alternative antigen for detecting *Brucella* IgG antibodies with high diagnostic accuracy [56]. These findings suggest that while Omp16 may serve as a supplementary antigen, other Omps or their combinations offer more reliable options for serodiagnosis.

A more advanced diagnostic approach involved the use of multiepitope recombinant proteins. In this study, major immunodominant epitopes from several outer membrane proteins, including Omp16, were selected and synthesized to construct a recombinant multiepitope outer membrane protein [57]. The resulting protein was expressed in *Escherichia coli* and tested in an indirect ELISA. The multiepitope protein demonstrated high sensitivity and specificity for detecting *Brucella* infections in both human and animal sera, offering a potential solution to the limitations of traditional whole-cell antigen-based assays [57]. The combination of Omp16 with other immunodominant epitopes such as Omp31 and Omp19 enhanced the diagnostic potential of this assay, suggesting that a multiepitope-based approach could provide better accuracy and reduce cross-reactivity.

Additionally, studies have focused on comparing the diagnostic efficacy of various *Brucella* outer membrane proteins, including Omp16, in identifying *Brucella*-positive sera. A comparative analysis of six recombinant *Brucella* outer membrane proteins—Omp10, Omp16, Omp19, Omp25, Omp31, and BP26—highlighted Omp16 as one of the key candidates for brucellosis diagnosis. The study revealed that Omp16 provided strong specificity and sensitivity in detecting *Brucella* infection in animal and human sera, especially in comparison to other antigens such as lipopolysaccharide (LPS) [55]. Omp16, alongside BP26, was shown to be highly effective in diagnosing human and goat brucellosis, with diagnostic accuracies reaching up to 95%. These findings emphasize the importance of Omp16 in serological diagnostics and its potential for use in routine diagnostic settings.

In addition to serological assays, although not commonly used in current diagnostic practice, earlier studies demonstrated that gene probes targeting Omp16 could differentiate between *Brucella* species and biovars through Southern blot hybridization. For example, the use of Omp16-based probes allowed the differentiation of *B. melitensis*, *B. ovis*, and *B. suis biovar* 2 from other *Brucella* species, providing useful insights for molecular epidemiology at the time [58]. However, with the advent of faster and more accurate methods such as real-time PCR and the Bruce-ladder multiplex PCR, these earlier techniques have largely been replaced in modern diagnostic workflows.

Previous studies have explored the diagnostic potential of Omp16 in bovine brucellosis, particularly in assessing its seroreactivity using techniques such as Western blotting. In one investigation, recombinant Omp16 demonstrated high specificity but low sensitivity when tested against *Brucella*-positive bovine sera [59]. While these findings suggest some immunoreactivity, the diagnostic utility of Omp16 as a standalone antigen appears limited. Given the subjective nature and limited scalability of Western blot, along with more recent comparative studies showing superior performance of other antigens such as BP26 and Omp31, Omp16 may serve better as a component in multivalent diagnostic approaches rather than as a primary target.

In conclusion, Omp16 has proven to be a valuable antigen in the diagnosis of brucellosis. Its versatility in various diagnostic formats, including indirect ELISA, multiepitope protein assays, and molecular techniques, positions it as a central component in advancing brucellosis diagnostics. The ongoing research and optimization of Omp16-based diagnostic tools will undoubtedly improve the accuracy, speed, and accessibility of brucellosis detection worldwide.

## 5. Future Directions and Conclusions

Future research on Omp16 should focus on elucidating the molecular mechanisms underlying its role in *Brucella* pathogenesis and reproductive pathology. While previous studies have demonstrated that Omp16 contributes to bacterial survival and immune modulation, the exact pathways and cellular targets involved remain insufficiently characterized. Specifically, understanding how Omp16 interacts with host cell receptors and modulates downstream signaling cascades will provide valuable insights into its function as a virulence factor. These findings could not only clarify the mechanisms of chronic *Brucella* infection but also identify novel therapeutic intervention points for controlling brucellosis.

Additionally, the development of high-resolution structural models of Omp16, either through X-ray crystallography, cryo-electron microscopy, or NMR, will deepen our understanding of its conformation and interaction with host molecules. Structural studies may reveal critical epitopes responsible for immune recognition or conserved motifs essential for membrane integration and stability. These data would also facilitate rational design of immunogenic peptides or Omp16-derived subunits suitable for vaccine formulation or diagnostic reagent development.

The potential of Omp16 as a diagnostic marker and vaccine antigen warrants continued and rigorous exploration. In diagnostics, although some early studies have reported promising results using recombinant Omp16 in ELISA formats, their limited sample sizes and lack of cross-validation across animal species and clinical populations have hampered wider application. Therefore, future studies should prioritize large-scale clinical validation of Omp16-based assays, with emphasis on multi-species evaluation including cattle, sheep, goats, and humans, comparison with established gold-standard tests, e.g., Rose Bengal, SAT, cELISA, and assessment of cross-reactivity with related bacterial infections. Diagnostic formats such as lateral flow assays or multiplex PCR incorporating omp16 could be particularly valuable for point-of-care testing in resource-limited or field settings.

In terms of vaccine development, Omp16 shows promise due to its strong immunogenicity and surface exposure, but current data suggest that its protective efficacy alone may be limited. Future work should explore Omp16 in combination with other immunodominant antigens such as BP26, Omp31, or L7/L12 to formulate multivalent subunit or DNA vaccines that elicit broad and robust immunity. Moreover, adjuvant selection, delivery platforms, and immunization regimens must be optimized to ensure safety and effectiveness, especially in vulnerable populations like pregnant animals or immunocompromised individuals.

In conclusion, Omp16 represents a multifaceted molecule at the intersection of *Brucella* virulence, host immune interaction, and translational application. Its roles in pathogenesis, diagnostic development, and immunoprophylaxis highlight its relevance in brucellosis research. While preliminary findings are promising, more comprehensive and mechanistically driven studies are essential to fully realize the potential of Omp16 in both veterinary and human medicine. A deeper understanding of this protein will significantly contribute to the control and eventual eradication of brucellosis.

## 6. Conclusions

Omp16 is a key outer membrane protein of *Brucella* that plays a critical role in the bacterium’s pathogenesis. Its involvement in reproductive pathology, including abortion and infertility, highlights its significance as a virulence factor and its potential as a target for diagnostic and therapeutic interventions. While significant progress has been made in understanding the structure and function of Omp16, further research is needed to fully harness its potential in controlling *Brucella* infections. As efforts continue to develop effective diagnostics and vaccines, Omp16 stands out as a promising candidate for inclusion in strategies aimed at reducing the burden of brucellosis in both humans and animals.

## Figures and Tables

**Table 1:** Omp16-based vaccine strategies against Brucella.

Vaccine Type	Antigen Composition	Animal Model	Immune Response	Protective Efficacy	Notes	Reference
Influenza viral vector	Omp16 or L7/L12	Pregnant heifers	Humoral + T-cell	Comparable to S19/RB51	Safe in pregnancy	[37]
Influenza vector + adjuvant	Omp16 + L7/L12 + Montanide/Chitosan	Cattle	Strong T-cell (CD4^+^/CD8^+^), IFN-γ	Superior to S19+adjuvant	Bivalent formulation	[38]
Influenza viral vector	Omp16 + L7/L12	Cattle (various ages)	Strong IgG response	60–75% in adults	Safe in pregnant cows	[39]
Influenza vector (Flu-BA_Omp19-SOD)	Omp16 + Omp19 + SOD	Sheep & goats	Th1-biased response	Partial (0–40%)	Lower in sheep	[40]
Influenza viral vector	Omp16 + L7/L12	Cattle	T-cell + long-term IFN-γ	Up to 71% protection	12 months post-boost	[41]
Flu-BA improved	Omp16 + L7/L12 + Omp19 + SOD	Pregnant sheep/goats	IgG2a, IFN-γ	55–66% in dams, 73–90% in offspring	Safe in pregnancy	[42]
Flu-BA	Omp16 + L7/L12	Sheep/goats	Strong T-cell (IFN-γ)	50–57% protection	No antibody detected	[43]
Recombinant protein (subunit)	Omp16 (U-/L-form)	Mice	CD4^+^, CD8^+^ T cells, IFN-γ	Comparable to S19	Oral/systemic routes tested	[23]
Self-adjuvanting	Unlipidated Omp16	Mice	Th1, dendritic activation	Comparable to S19	Oral immunization possible	[17]
DNA vaccine	Omp16 + L7/L12	Mice	IgG2a, T-cell, IFN-γ	Higher than monovalent DNA	Divalent DNA construct	[44]
Recombinant protein	rOmp16	Mice	IgG1/2a, IFN-γ, IL-4	Significant protection	Against B. abortus & B. melitensis	[25]

## Data Availability

No new data were created or analyzed in this study. Data sharing is not applicable to this article.
